# The level of response to alcohol is related to level of se-phosphate in the general population

**DOI:** 10.1093/alcalc/agaf065

**Published:** 2025-10-30

**Authors:** Jørgen G Bramness, Jørg Mørland, Jenny Moe, Susmita Pandey, Knut Ragnvald Skulberg, Ingeborg Bolstad

**Affiliations:** Section for Clinical Addiction Research, Oslo University Hospital, Post Box 4959 Nydalen, 0424 Oslo, Norway; Norwegian Institute of Public Health, Post Box 222 Skøyen, 0213 Oslo, Norway; Research Center for Substance Use Disorders and Mental Illness, Innlandet Hospital Trust, Post Box 104, 2381 Brumunddal, Norway; Institute of Clinical Medicine, University of Tromsø—The Arctic University of Norway, Hansine Hansens veg, 9019 Tromsø, Norway; Research Center for Substance Use Disorders and Mental Illness, Innlandet Hospital Trust, Post Box 104, 2381 Brumunddal, Norway; University of Inland Norway, Post Box 400 Vestad, 2418 Elverum, Norway; Research Center for Substance Use Disorders and Mental Illness, Innlandet Hospital Trust, Post Box 104, 2381 Brumunddal, Norway; Institute of Clinical Medicine, University of Tromsø—The Arctic University of Norway, Hansine Hansens veg, 9019 Tromsø, Norway; Research Center for Substance Use Disorders and Mental Illness, Innlandet Hospital Trust, Post Box 104, 2381 Brumunddal, Norway; Institute of Clinical Medicine, University of Oslo, Post Box 1171, 0318 Oslo, Norway; Norwegian Institute of Public Health, Post Box 222 Skøyen, 0213 Oslo, Norway; Institute of Clinical Medicine, University of Oslo, Post Box 1171, 0318 Oslo, Norway

**Keywords:** alcohol, level of response, serum phosphate

## Abstract

**Background:**

A low subjective response to alcohol predicts increased consumption and risk for alcohol use disorder (AUD). While self-report tools like the self-rated effects of alcohol (SRE) questionnaire assess this, biological correlates remain understudied. This study investigates the relationship between SRE scores, serum phosphate (se-P), and other alcohol related measures using data from one experimental and two clinical cohorts.

**Material and methods:**

Participants from three cohorts completed the SRE questionnaire. Blood samples were collected to measure alcohol use biomarkers and other blood measures like se-P. Statistical analyses assessed relationships between SRE scores and biochemical measures across groups.

**Results:**

AUD patients had higher SRE lately scores (mean 6.74) than blood donors (3.08), and higher se-P (1.23 mmol/L vs. 1.05). SRE scores correlated positively with se-P in both the experimental group (r = 0.507, *P* = .027) and blood donors (r = 0.377, *P* = .011), but not in AUD patients. Prolactin correlated with SRE scores where measured.

**Discussion:**

This study confirmed a positive correlation between se-P and SRE scores in non-AUD groups, possibly supporting a link between se-P and presystemic alcohol metabolism. No such relationship was found in AUD patients, possibly due to nutritional supplementation of phosphate and other nutrients. The observed association between prolactin and SRE in both observational groups was unexpected. Overall, these findings further indicate that se-P may play a role in alcohol metabolism, especially presystemic alcohol metabolism, but further research and replication are needed to clarify mechanisms.

## Teaser

**Short Summary:** A low subjective response to alcohol predicts higher consumption and alcohol use disorder (AUD). A low subjective response scores correlated with increased serum phosphate (se-P) in non-AUD groups, but in not AUD patients. Findings suggest se-P involvement in presystemic alcohol metabolism, though nutritional factors may obscure associations in AUD.

## Introduction

Studies have shown that a person’s sensitivity to the acute effects of alcohol is one of several endophenotypes that influence the risk of heavy drinking and of developing alcohol problems (Heath et al. 1999, Schuckit and Smith 2000, Schuckit et al. 2019). Research on this subjective response to alcohol has been performed experimentally by giving participants alcohol and measuring their reactions; a low response to alcohol with few negative acute effects cause a tendency to increase alcohol consumption (Boyd and Corbin 2018), and moreover, it seems to predict future alcohol use disorder (AUD) (Schuckit 1994).

Response to alcohol can be measured experimentally, by administering alcohol and monitoring effects. This is, however, laborsome and costly. Self-report instruments, asking people retrospectively about their experience with alcohol inebriation and intoxication have also been developed and have proven useful (Schuckit et al. 1997a). One such questionnaire is self-rating of the effects of alcohol (SRE) that has been found to tap into the same information as found by subjective effects of an alcohol challenge (Schuckit et al. 1997b, Schuckit et al. 2009). It has also shown a good ability to predict negative effects of alcohol (Piasecki et al. 2012) and future AUD (Schuckit et al. 2008).

Despite the many studies performed on the different aspects of low level of response to alcohol and the SRE instrument, few studies utilizing biological measures have been performed. In a previous study we found that the ability to take more alcohol before feeling its effects was partially explained by the alcohol not reaching systemic circulation, and that SRE was related to prehepatic and probably gastric (hereafter presystemic) alcohol metabolism (Bramness et al. 2023).

Presystemic alcohol metabolism is difficult to measure other than in an experimental setup, often measuring the difference between oral and parenteral administration of equivalent doses of alcohol (Frezza et al. 1990). We have however, in another previous study found that presystemic alcohol metabolism is closely related to circulating levels of serum phosphate (se-P) (Bramness et al. 2021). The difference between the highest and lowest maximum blood alcohol concentration in that study was as much as 30%–40% and correlated negatively to se-P levels. The results suggested that phosphate plays a role in prehepatic, possibly gastric, alcohol metabolism. The mechanism behind this is unclear and the finding has not yet been replicated. It would, however, be of value to have a biological measure related to presystemic alcohol metabolism.

In the current study we wanted to further investigate the relationship between SRE, se-P and presystemic alcohol metabolism. We utilized data from two different studies, one experimental study with 20 healthy volunteers that our previous findings are taken from (Bramness et al. 2021, Bramness et al. 2023) and one clinical observational study of 113 AUD patients and 70 healthy blood donors. In both these studies we asked about response to alcohol using the SRE questionnaire and measured se-P. We hypothesize that there is a relationship between se-P and level of response to alcohol as measured by SRE.

## Materials and methods

### Study participants and design

In the current study we included participants from three data sources. Firstly, we included 20 young adult male healthy volunteers who participated in a controlled study on the relationship between se-P and alcohol elimination (Bramness et al. 2021, Bramness et al. 2023). These healthy volunteers were recruited through an open advertisement in the University College of Norwegian Correctional Service. Inclusion criteria were male gender and Caucasian origin with at least one experience with drinking more than five units of alcohol at least one occasion in the past. Exclusion criteria were significant medical illness, alcohol or other substance use disorders, and metabolic disorders. Their average age (standard deviation) was 28.9 (5.3) years.

Secondly, 69 healthy individuals recruited from a blood donor center were included (Bolstad et al. 2023). Inclusion criteria were passing screening for blood donation. The group consisted of 25 (36%) females and had the average age 46.3 (12.7) years.

Thirdly, we collected data from three AUD rehabilitation clinics. The material has been described in a previous publication (Bolstad et al. 2021). The clinics offered long-term residential treatment stays (>30 days) and patients were considered for inclusion in the study if they had current AUD as diagnosed according to International Classification of Diseases 10th Revision (ICD-10). They were not included if the clinical staff judged that they were not fit due to severe somatic illness, psychosis, cognitive impairment, or inability to speak a Scandinavian language. Of the 366 patients who were admitted to treatment in the clinics during our inclusion period from January 2018 to March 2019, 224 (61%) were considered eligible for participation in this study. Eligible participants were provided with information about the study and of these, 113 (51%) patients signed informed written consent and were enrolled in the study. The final group included 30 (27%) females and had an average age of 51.1 (10.6) years. The participants had been abstinent from alcohol a minimum of 5 days, with a median of 19 days before enrolment in the study.

### Measured variables

From all the subjects the following data was gathered: background variables: age (years) and body mass index (BMI) (kg/m2); alcohol related parameters: Alcohol Use Disorders Identification Test (AUDIT) score, B-phosphatidylethanol (PEth) (μmol/L), carbohydrate deficient transferrin (CDT) (%), γ-glutamyl transpeptidase (γGT) (U/L), aspartate aminotransferase (ALAT) (U/L), alanine aminotransferase (ASAT) (U/L), mean corpuscular volume (MCV) (fL), and prolactin (mU/L); other biochemical measures: c-reactive protein (CRP) (mg/L), ferritin (μg/L), vitamin B12 (pmol/L), vitamin B9 (folic acid) (nmol/L), vitamin D3 (25-OH-vit D) (nmol/L), calcium (mmol/L), phosphate (mmol/L), cholesterol (mmol/L), high density lipoprotein (HDL) (mmol/L), and low-density lipoprotein cholesterol (LDL) (mmol/L). PEth, ASAT, prolactin, ferritin, vitamin B12, vitamin B9, calcium, cholesterol, HDL, and LDL were not available for the experimental group. Blood samples were drawn in relationship to answering the questions on level of response to alcohol.

Level of response or subjective effects of alcohol were measured by the SRE, which is a twelve item questionnaire asking how many units of alcohol a person had to drink to feel any effect, produce dizziness or slurred speech, be associated with a stumbling gait or to have contributed to falling asleep at three points in time (Schuckit et al. 1997a). These times are when first starting to drink (SRE early), during the last period when drinking at least once a month (SRE lately) and in periods of heavy drinking (SRE heavy). The SRE has mostly been used in English speaking countries but was for the purpose of earlier study translated into Norwegian (Bramness et al. 2021). For the analysis in this report, we used the first question (how many drinks to feel any effect of alcohol) of the SRE lately component, as there was relatively high nonresponse to the higher hierarchical questions (concerning dizziness or slurred speech). We also used the same question of the SRE early.

### Statistical analyses

The statistical analysis was performed using IBM SPSS Statistics for Windows version 25 (IBM Corp., Armonk, NY). Bivariate statistical tests were done by using Student’s *t*-test for comparing continuous variable across two groups or Pearson’s r correlation for looking at the relationship between two continuous variables. Exact p-values are given.

### Ethics

All research was done according to the Helsinki declaration of medical research. Prior to inclusion, written informed consent was obtained from participants in all three groups. All participants in the study were fully entitled to withdraw their consent at any time during the study and were duly informed of this. For the first group of subjects there was approval from the Norwegian Regional Ethics Committee (REK case ref. 2013/1563). These healthy volunteers were insured as part of the project leader’s membership with the Norwegian Drug Liability Association (ref. 5041916/1) covering incidents in relation with the experiment. They received bank transfer amounting Norwegian Kroner 2000 (approximately € 200) in compensation for the time participating in experiment and taxi fare was paid to return home after the experiment. In the observational study of the patients treated for AUD and of the blood donor group we had a concomitant approval from the Norwegian Regional Ethics Committee (REK case ref. 2017/1314). Participants in these two groups were not compensated for their participation.

## Results

Table 1 shows the background variable, alcohol use measures and baseline medical biochemistry parameters in three groups. The experimental group consisted of only young men who had a slightly lower BMI than the AUD patients and blood donor group. Furthermore, the young males in the experimental group reported an AUDIT score as high as 9.22 (3.25), between the blood donor group (*P* < .001) and the AUD group (*P* < .001). The AUD group had higher levels of PEth and γGT, but not CDT. But the experimental group had higher levels of CDT (*P* = .038). The AUD group also had a higher level of CRP and of most nutritional elements, except vitamin D3 which was lower (*P* < .001). The experimental group had a higher se-P measure (1.30; 0.19) than the blood donor group (1.04; 0.17) (*P* < 0.001), and the AUD group (1.14; 0.21) (*P* = .005), the AUD group also being higher than the blood donor group (*P* = .012).

**Table 1:** Baseline characteristics of the three groups investigated groups. Measures are given as mean (standard deviation) or as N (%).

			Experimental group	Blood donors	AUD group	Post hoc *P*-values		
		*n*	*N* = 19	*N* = 45	*N* = 63	1 vs. 2	1 vs. 3	2 vs. 3
Background variables								
	Age (years)	127	29.00 (5.37)	46.78 (11.94)	53.09 (9.46)	**<.001**	**<.001**	**.003**
	Gender (N (%) females)	127	0 (0)	13 (29)	17 (27)	**.009**	**.011**	.828
	BMI (kg/m^2^)	127	25.45 (2.50)	27.71 (4.64)	27.49 (4.82)	.050	.082	.806
Alcohol-related parameters								
	AUDIT score	126	9.22 (3.25)	4.56 (3.11)	28.62 (6.44)	**<.001**	**<.001**	**<.001**
	SRE early (units)	120	2.79 (1.18)	2.21 (1.01)	3.05 (2.14)	.053	.615	**.019**
	SRE lately (units)	127	4.53 (1.81)	3.08 (1.48)	6.66 (5.26)	**.001**	.087	**<.001**
	PEth (μmol/L)	105	n.a.	0.11 (0.27)	0.41 (0.43)			**<.001**
	CDT (%)	126	1.43 (2.01)	0.76 (0.45)	1.08 (1.02)	**.038**	.314	.055
	γGT (U/L)	126	28.11 (11.17)	21.40 (12.81)	91.69 (131.55)	.054	**.039**	**.001**
	ALAT (U/L)	126	33.95 (9.11)	24.36 (11.68)	42.98 (36.85)	**.002**	.295	**.001**
	ASAT (U/L)	107	n.a.	22.13 (6.48)	32.27 (22.56)			**.004**
	MCV (fL)	126	88.74 (3.90)	96.89 (4.24)	97.16 (7.17)	**<.001**	**<.001**	.822
	Prolactin (mU/L)	107	n.a.	148.58 (65.88)	145.24 (75.16)			.812
Other biochemical measures								
	CRP (mg/L)	126	1.71 (3.57)	1.36 (1.21)	4.66 (8.93)	.553	.165	**.015**
	Ferritin (μg/L)	107	n.a.	61.31 (28.21)	155.89 (16.22)			**<.001**
	Vitamin B12 (pmol/L)	105	n.a.	304.58 (77.67)	417.02 (152.14)			**<.001**
	Vitamin B9 (pmol/L)	100	n.a.	13.17 (7.31)	21.12 (11.53)			**<.001**
	Vitamin D3 (nmol/L)	107	n.a.	84.87 (2.05)	65.77 (25.60)			**<.001**
	Calcium (mmol/L)	98	n.a.	2.31 (0.06)	2.34 (0.09)			.096
	Phosphate (mmol/L)	117	1.30 (0.19)	1.04 (0.17)	1.14 (0.21)	**<.001**	**.005**	**.012**
	Cholesterol (mmol/L)	66	n.a.	4.81 (0.74)	5.31 (1.09)			**.033**
	HDL (mmol/L)	66	n.a.	1.30 (0.36)	1.35 (0.43)			.621
	LDL (mmol/L)	66	n.a.	3.10 (0.70)	3.58 (1.11)			**.040**

Significant values (*P* < .05) are shown in bold. Abbreviations: AUDIT, alcohol use disorder identification test; CDT, carbo deficient transferrin; γGT, gamma glutamyl transferase; LDL, low density lipoprotein; n.a., not analyzed; U, units.

Concerning the number of drinks needed to feel any effect when starting to drink (SRE early), the AUD group scored slightly higher than the blood donor group (*P* = .019). The differences were more pronounced for the number of drinks needed to feel an effect of alcohol lately, with a mean score of 6.66 (5.26) among the AUD patients being higher than the blood donor group [3.08 (1.48) (*P* < .001)], but quite similar to the number of drinks needed in the experimental group [4.53 (1.81) (*P* = .087)].

Table 2 gives a correlation matrix between the number of drinks lately needed to feel any effect in all the three researched groups. This SRE score correlated positive with BMI in the AUD group 0.266 (0.035) and with ALAT in the experimental group −0.457 (0.049). Prolactin was only measured in the AUD and blood donor groups but correlated positively with SRE in both groups. Prolactin did not correlate significantly with neither amount of alcohol taken on a regular basis (measured by AUDIT) or any of the biological alcohol use parameters (such as CDT and γGT) (data not shown in table). Also, prolactin did not correlate with se-P. All these correlations were checked in both the AUD patient group and in the blood donor group, and even when divided by gender.

**Table 2:** Correlates to the self-reported number of drinks needed to feel any effect lately (SRE). Measures given in Pearson’s r with *P*-value.

		Experimental group		Blood donors		AUD group	
		*N* = 19		*N* = 45		*N* = 64	
		r	*P*-value	r	*P*-value	r	*P*-value
Background variables							
	Age (years)	−0.423	.071	−0.167	.273	0.091	.478
	BMI (kg/m^2^)	−0.071	.772	0.013	.931	0.266	**.035**
Alcohol related parameters							
	AUDIT score	0.355	.148	0.079	.606	0.049	.703
	PEth	n.a.		0.116	.453	−0.068	.605
	CDT	−0.390	.098	0.140	.358	0.125	.333
	γGT	−0.163	.505	−0.057	.709	−0.158	.219
	ALAT	**−0.457**	**.049**	0.032	.836	−0.089	.494
	ASAT	n.a.		−0.067	.661	−0.064	.620
	MCV	0.360	.130	0.043	.782	0.044	.731
	Prolactin	n.a.		**0.315**	**.035**	**0.437**	**<.001**
Other biochemical measures							
	CRP	−0.035	.886	0.035	.820	0.003	.984
	Ferritin	n.a.		−0.002	.988	−0.144	.264
	Vitamin B12	n.a.		−0.099	.518	−0.110	.401
	Vitamin B9	n.a.		0.108	.485	0.011	.935
	Vitamin D3	n.a.		0.161	.292	−0.17	.186
	Calcium	n.a.		0.165	.279	0.187	.180
	Phosphate	**0.507**	**.027**	**0.389**	**.008**	−0.125	.372
	Cholesterol	n.a.		−0.052	.737	−0.319	.159
	HDL	n.a.		−0.048	.756	−0.022	.924
	LDL	n.a.		0.021	.889	−0.263	.250

Significant values (*P* < .05) are shown in bold.

**Figure 1:**
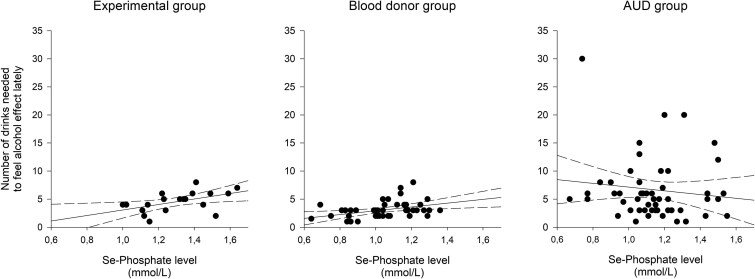
The relationships between se-phosphate levels (in mmol/L) of the three included samples and subjectively reported number of drinks needed to feel an effect of alcohol during the last three months. There was a positive and significant relationship found in the experimental group (Pearson’s *r* 0.507; *P* = .027; r^2^ = 0.257) and the blood donor group for AUD study (Pearson’s *r* 0.389; *P* = .008; r^2^ = 0.151), but not among the AUD patients (Pearson’s *r* − 0.125; *P* = .372; r^2^ = 0.016).

Se-P levels correlated highly and positively with SRE score in both the experimental group (Pearson’s r = 0.507; *P* = .027) and the blood donor group (0.389; 0.008), but not in the AUD group (−0.125; 0.372). We also performed sensitivity analysis, taking out the extreme values for number of drinks needed to feel any effect and analysing gender specific without this changing our results substantially. This is also illustrated in Fig. 1. Se-P did not show any relationship to SRE early (data not shown in table).

## Discussion

The major findings in this study collecting data from three different groups was a positive correlation between se-P levels and response to alcohol as measured by SRE in the two non-AUD groups. We thus confirmed our hypothesis of there being such a relationship.

We have earlier shown that se-P level seems to be related to presystemic alcohol metabolism (Bramness et al. 2021). Why se-P is related to presystemic alcohol metabolism has not been established. In reality, very little is known about the role of phosphate in alcohol use and metabolism, except for some studies exploring phosphate’s role in alcohol induced pancreatitis (Farooq et al. 2021, Pandol 2022) and that severe hypophosphatemia may play a role in morbidity of end stage AUD (Knochel 1980), possibly due to renal phosphate loss (Angeli et al. 1991). Related to this, presystemic alcohol metabolism seems to be dramatically reduced in end-stage AUD (Frezza et al. 1990), indirectly pointing to a relationship between se-phosphate and presystemic alcohol metabolism.

Our previous study showed that SRE is related to presystemic alcohol metabolism (Bramness et al. 2023), but the current study is the first to show a direct relationship between SRE and se-P levels. The relationships between presystemic alcohol metabolism and SRE (Bramness et al. 2023) and se-P (Bramness et al. 2021) were based on data from a single experimental study of a rather small group of health young male adults. To demonstrate the direct relationship SRE and se-P in the original group and to replicate this is in a different group (here healthy blood donors) is thus of high value, further strengthening the hypothesis that phosphate may play a role in presystemic alcohol metabolism, but of reasons yet to be established (Bramness et al. 2021).

In the current study we observed higher se-P levels in the experimental group compared to the blood donor group. Other investigations have shown that se-P varies with age and gender, with younger males having higher levels, but gender differences seem to diminish over time (Bosman et al. 2024). This could explain the higher values and larger spread found among the blood donor group consisting of blood donors of both sexes and of a higher age.

No systematic relationship between se-P levels and SRE was found in the AUD group. They also had a much higher spread of their se-P. Patients with AUD are often given nutritional supplements. As the wide variation also is evident in the other trace elements and vitamin measured, this is the most likely explanation. Also, some of the AUD patients reported extreme values of how many units of alcohol they must consume to feel any effect, possibly raising a suspicion of misunderstanding the SRE questionnaire. But it is also known that some people with AUD can consume extreme amounts of alcohol without being inebriated (Elvig et al. 2021).

There was also a close relationship between SRE and se-prolactin levels both among the blood donors and AUD patients. The release of prolactin, to a major extent, is inhibited by the neurotransmitter dopamine released in the hypothalamus (Ben-Jonathan and Hnasko 2001, Fitzgerald and Dinan 2008). To this end, the level of prolactin in the peripheral blood may be viewed as a reflection of the hypothalamic dopaminergic activity. Studies have shown that in addition to chronic heavy alcohol use, prolactin may also elevate in acute alcohol intoxication (Frias et al. 2002), alcohol withdrawal and early abstinence (Wilhelm et al. 2011). The elevation of prolactin during alcohol withdrawal is associated with the severity of alcohol dependence and withdrawal symptoms (Wilhelm et al. 2011, Pandey et al. 2021) and higher craving (Hillemacher et al. 2006). Such connotations may be expected in patients recovering from AUD and then being related to the level of their drinking. More surprising is that this relationship was also found among those consuming low or moderate amounts of alcohol.

The AUD patient group reported a higher SRE score when starting to use alcohol compared to the other groups. This is in line with the well-known fact that a low level of response to alcohol when starting drinking is a predictor for AUD (Schuckit 1994). The AUD group had higher PEth levels and levels of other alcohol related biochemical compared to the blood donor group. However, CDT was similar in the three groups. Even if CDT is a recognized marker of prolonged elevated alcohol use, it tends to normalize within 2–3 weeks, corresponding with the abstinence period of the studied group (Arnts et al. 2021). Lastly, the AUD had been given substantial oral nutritional supplements of vitamins and trace metals, including magnesium and phosphate. This may be the explanations for quite a few observations being over reference areas for several compounds. All this made the investigation of these elements in the AUD group difficult to interpret and may at least partially explain the lack of relationship between se-P and SRE among the AUD patients.

### Limitations

The current findings stem from three different groups, that partook in an experiment or were observed for other purposes. We build our conclusion on a small number of findings and these need to be replicated. Some parameters lacked in some groups, making the comprehensiveness of the data suboptimal, and especially the lack of the highly sensitive and specific alcohol measure PEth in the experimental group should be noted. Also, for practical reasons, only males were included in the experimental study, and it is unknown how these measures would be in females. Lastly, the AUD group was investigated some week after detoxification. In this period, they had recovered from some of the effects of alcohol use and withdrawal and had received large vitamin and nutritional supplements according to common practice (ref), making the correlation analysis difficult to interpret. Future research should try to include patients at an earlier time point and try to amend also other weaknesses of the current study.

### Conclusion

The current study shows that a low level of response to alcohol (as measured by a high SRE score) is negatively related to se-Pin individuals not in treatment for AUD. This finding indirectly strengthens the assumption that presystemic alcohol metabolism is related to an individual’s se-P and that there is a relationship between low level of response to alcohol and high degree of presystemic alcohol metabolism and at least among the participants that were not in treatment for their AUD. Further investigations are needed to corroborate these findings or even find out what the mechanism behind this finding is.

## Data Availability

The data that support the findings of this study are not publicly available. Data are, however, available from the authors upon reasonable request and with permission from the Norwegian Ethical Review Board.

## Notes

*Conflict of interest:* Knut Ragnvald Skulberg was previously involved in the commercial firm Primo Inc. which produced the sports drink ‘c-Foz’ who paid for the experimental part of this study with an unrestricted grant. Professor Jørgen G. Bramness was paid a one-time advisory honorarium in 2014 of 1000, Norwegian Kroner (approximately € 1100) from Primo Inc. for work on designing the project. The results of the current study were taken from the placebo arm of a study designed to investigate c-Foz effect on hang-over, with negative results. The observational study of AUD patients and healthy blood donors were paid for the Research Council of Norway (grant number FRIPRO 251140). None of the authors have any conflicts of interest to report that could inflict on the current study.
